# Natural History of Mandibular Function in Spinal Muscular Atrophy Types 2 and 3

**DOI:** 10.3233/JND-240007

**Published:** 2024-04-30

**Authors:** H. Willemijn van Bruggen, Camiel A. Wijngaarde, Faylynn Asselman, Marloes Stam, Nico H.J. Creugers, Renske I. Wadman, W. Ludo van der Pol, Stanimira I. Kalaykova

**Affiliations:** a Department of Dentistry, Radboud University Nijmegen Medical Center, Nijmegen, The Netherlands; b Department of Neurology and Neurosurgery and Spieren voor Spieren Kindercentrum, UMC Utrecht Brain Center, University Medical Center Utrecht, Utrecht University, Utrecht, The Netherlands

**Keywords:** Spinal muscular atrophy, bulbar function, dysphagia

## Abstract

**Background:**

Hereditary proximal spinal muscular atrophy (SMA) is characterized by abnormal alpha motor neuron function in brainstem and spinal cord. Bulbar dysfunction, including limited mouth opening, is present in the majority of patients with SMA but it is unknown if and how these problems change during disease course.

**Objective:**

In this prospective, observational, longitudinal natural history study we aimed to study bulbar dysfunction in patients with SMA types 2 and 3.

**Methods:**

We included 44 patients with SMA types 2 and 3 (mean age was 33.6 (95% CI 28.4;38.9) and re-examined them after on average 4 years. None were treated with *SMN-*modulating treatments before or during the course of this study. Longitudinal assessments included a questionnaire on mandibular and bulbar function, the Mandibular Function Impairment Questionnaire (MFIQ), and a clinical examination of masticatory performance, maximum voluntary bite force, and mandibular movements including the active maximal mouth opening.

**Results:**

We found significant higher MFIQ scores and a significant decrease of all mandibular movements in patients with SMA type 2 (*p* < 0.001), but not in SMA type 3. Masticatory performance and maximum voluntary bite force did not change significantly. Mean reduction of active maximal mouth opening at follow-up was 3.5 mm in SMA type 2 (95% CI: 2.3; 4.7, *p* < 0.001). SMA type 2 was an independent predictor for a more severe reduction of the mouth opening (β= –2.0 mm (95% CI: –3.8; –0.1, *p* = 0.043)).

**Conclusions:**

Bulbar functions such as mandibular mobility and active maximum mouth opening decrease significantly over the course of four years in patients with SMA type 2.

## INTRODUCTION

Hereditary proximal spinal muscular atrophy (SMA) is caused by loss of function of the *SMN1* gene [1]. The lack of SMN protein production causes degeneration of alpha motor neurons in the spinal cord. This leads to generalized weakness of predominantly proximal limbs, axial and respiratory muscle groups [1]. Brainstem motor nuclei also become affected, resulting in weakness of the tongue and muscles for mouth closure and opening [1–5]. This contributes to significant impairments in daily life, e.g. with eating and swallowing, but may also complicate dental and medical procedures [3–9].

When asked, patients with SMA often report bulbar dysfunction [10]. A small number of studies addressed prevalence and severity of limitations of mastication, biting force and mandibular mobility [4, 5, 7, 9, 11]. Decreased mandibular mobility, that is reflected by decreased active maximal mouth opening (AMMO), is probably the best-studied bulbar function in SMA. We previously concluded from cross-sectional data that mandibular mobility is limited in patients with SMA types 1c and 2, but not in type 3 [4, 5]. This is caused by fatty infiltration of the lateral pterygoid muscles. Its relevance and severity are illustrated by the finding that it may interfere with intubation at a relatively early age [4, 5, 9]. Cross-sectional data also suggest that these limitations progress over the years, but there is no longitudinal data available on the natural history of AMMO or any other feature of bulbar weakness in patients with SMA.

Survival and natural history studies of motor function in SMA have facilitated clinical trial design to evaluate efficacy of genetic therapies for SMA. The lack of studies on the natural course of bulbar function impairs efficacy analyses at the level of the brainstem. The objective of this study was therefore to document changes of bulbar dysfunction in patients with SMA, using questionnaires and functional examinations, including maximum mouth opening.

## MATERIAL AND METHODS

### Design and Participants

Patients enrolled in this study are participating in an ongoing prospective cohort study on SMA in The Netherlands [12]. The local Medical Ethics Committee approved our study (No. 09-307/NL29692.041.09). Written informed consent was obtained from all participants and/or their parents in case of minors. Sixty patients with SMA types 2 and 3, who participated in our baseline study between 2013 and 2014, were invited for re-examination between December 2017 and July 2018 [5]. Patient characteristics and medical history data were retrieved from the national SMA database [12]. None of the patients were treated with *SMN-*modulating treatments before or during the course of this study.

For all patients we used multiplex ligation-dependent probe amplification (MLPA; SALSA kit P021-B1-01, MRC-Holland) to confirm loss of *SMN1* function and to determine *SMN2* copy numbers. We distinguished SMA types based on age at symptom onset and acquired motor milestones, following the SMA consortium criteria [13–15].

### Questionnaires

We used a screening questionnaire to assess pain characteristics in the head and neck region, as well as mandibular function, and the ‘Mandibular Functional Impairment Questionnaire’ (MFIQ) to assess difficulties in performing a particular mandibular function, e.g., chewing or yawning [16–19]. The MFIQ consists of 17 questions, which can be subdivided into functional capacity and feeding. It uses a five-level Likert scale ranging from ‘0’ (no difficulty) to ‘4’ (very difficult or impossible without help). We used sum scores of all items combined for analyses (S, range 0–68). A higher score indicates more perceived mandibular function impairments and a MFIQ score of ‘0’ indicates no impairment in mandibular functioning [19].

### Structured patient interviews

Patients were asked if they experienced problems with feeding and swallowing. A feeding disorder was defined as avoidance of certain foods or problems with mastication. Mastication difficulties were defined as difficulties during mastication of hard, sticky or soft food. Additional questions were if patients experienced problems during feeding with opening the mouth, chewing food and if food adaptation was necessary. A swallowing disorder (or dysphagia) was defined as a disorder in the oral, the pharyngeal or esophageal phase of the swallowing process, i.e. moving food or fluids from the oral cavity to the throat or delayed passage of food or fluids through the esophagus. We used regular blockage of the throat by food or drinks as a definition for choking (Table 1) [3].

**Table 1:** Table1

	Baseline study (2014)	Follow-up study (2018)	Lost to follow-up^a^
Patients, *n*	44	44	16
Gender (F:M)	25 : 19		8 : 8
SMA type 2	22 (50%)		11 (69%)
SMA type 3a	12 (27%)		4 (25%)
SMA type 3b	10 (23%)		1 (6%)
Age, years (mean, range)	33.4 (8.9–70.2)	37.6 (12.4–74.0)	29.3 (8.7–65.7)
Disease duration, years (mean, range)	26.6 (4.4–64.8)	30.8 (7.9; 68.6)	24.5 (4.4; 57.3)
Scoliosis surgery	23 (52%)	26 (59%)	9 (56%)
Dysphagia^b^	18 (41%)	27 (62%)	7 (44%)
Ventilatory support
None	38 (85 %)	38 (85%)	9 (56%)
Part-time (nocturnal)^c^	2 (5 %)	0	0
Continuous noninvasive	2 (5 %)	4 (10%)	6 (38%)
Continuous + tracheostomy	2 (5 %)	2 (5%)	1 (6%)

SMA = spinal muscular atrophy; ^a^Lost to follow up from original cohort of 60 patients [5]. Reasons for lost to follow up includes: no response (*n* = 5), not willing to participate (*n* = 7) and passed away (*n* = 4). ^b^Dysphagia: Patients with a score > 1 have complaints of dysphagia, including choking on solid foods or the feeling of food not passing the throat after swallowing. ^c^Ventilatory support Part-time (nocturnal): ≤8 hour suppor. Participant characteristics at baseline and after 4 years.

### Clinical examination

**Fig. 1:**
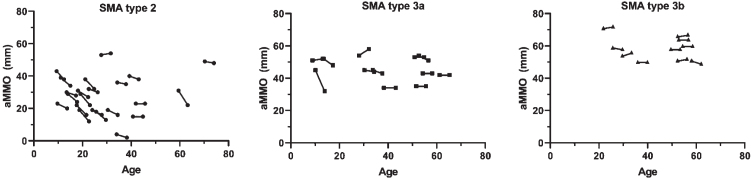
Correlation between age and active mouth opening in patients with SMA type 2, 3a and 3b. Active maximum mouth opening (AMMO) measured in mm is significantly decreased at follow-up in patients with SMA type 2, but not SMA types 3a and 3b.

All patients underwent a detailed clinical examination of the masticatory system, using a validated procedure [16, 20]. We assessed mandibular movements with patients in an upright and neutral position. A metal ruler was used to measure the AMMO, left and right lateral range of motion (ROM), protrusion, the vertical overlap of the front teeth in closed mouth position (overbite) and the horizontal overlap (overjet). The distance between central incisal edges combined with overbite was defined as AMMO (Fig. 1). We encouraged participants verbally during measurements. In a previous study we showed that 144 healthy controls had an AMMO of >35 mm. Therefore, we defined a limited AMMO as ≤35 mm [4]. The masseter and temporalis muscles and the lateral pole of the mandibular condyle of the temporomandibular joint (TMJ) in an open and closed position were palpated with the index finger [21]. A numerical rating scale (NRS), with scores ranging from 0–10, was used to document pain throughout the clinical examination [22]. During traction and translation of the mandibular condyle, the sliding capacity of the TMJ was assessed. The sliding capacity was defined as normal when the condyle was sliding to and beyond the crest of the articular eminence and limited when there was an absence or severe impairment of sliding of the lateral pole of the mandibular condyle. The number of occlusal contacts between the premolar and molar teeth of the upper and lower jaw recorded with wax plates (Moyco beauty pink plate wax, 2270 g). Perforations in the wax plates indicated the presence of occlusal contacts (OC-score, maximum score unilaterally 5 and bilaterally 10) between upper and lower jaw premolar and molar teeth [5].

### Measurement of the anterior maximum voluntary bite force

The maximum voluntary bite force (MVBF) was measured between the maxillary and mandibular anterior teeth with the “Vrije Universiteit Bite Force Gauge” (VU-BFG) [23]. This handheld device measures bite force in kilograms (range 0–50 kilograms) using a loaded cell (LPM 510 250 lb). The bite force transducer was calibrated before and after final measurements. Patients were requested to apply maximum bite force for 3 seconds with the strain gauge between their anterior teeth. This test was performed three times and the highest outcome was recorded.

### Masticatory performance: mixing ability test

We measured masticatory performance using the mixing ability test [24, 25]. For this test, patients chewed 20 strokes on a two-colored wax tablet offered at room temperature (20°C). To measure the amount of mixing, the chewed wax was sandwiched and optically scanned using a high-quality scanner (Epson V750, Long Beach, California). The images of the wax were processed using Adobe Photoshop, CS3 extended (Adobe, San Jose, California) [24]. The degree of mixing ability of the colored layers on both sides was quantified by calculating the Mixing Ability Index (MAI). A badly mixed wax tablet leads to a high MAI, indicating a low mixing performance, a well-mixed wax tablet leads to a low MAI, indicating a high mixing performance [24].

### Data availability

Anonymized data that support the findings of this study are available from the corresponding author on reasonable request.

## STATISTICS

Continuous data are presented as mean and 95% confidence intervals (CI), whereas categorical data are presented as number and percentage. Continuous data were reviewed visually for a normal distribution.

To assess changes in outcome variables over time we used the paired *t*-test or McNemar test to compare results between first and follow-up measurement.

We used univariate linear regression to identify factors associated with an AMMO decrease during follow-up. Factors investigated included SMA type, age, disease duration, AMMO, MVBF, mixing ability, and MFIQ at first measurement. Factors with a significant association were combined in a multivariable model using forward stepwise selection to identify the strongest independent predictors of a decrease of AMMO over time. Outcomes of regression analyses are presented as unstandardized β, 95% CI and *p*-value. All statistical analyses were performed using IBM SPSS Statistics 25 statistical software (SPSS Inc., Chicago, IL). The level of significance was set at *p* = 0.05.

## Results

At follow up 44 out of the original sample of 60 patients (73%) with SMA types 2 and 3 were enrolled into this study [6]. Baseline characteristics are summarized in Table 1. Sixteen patients were lost to follow up (no response: *n* = 5, not willing to participate: *n* = 7 and passed away: *n* = 4).

Relatively more patients with SMA type 2 declined enrollment (69%) compared to patients with type 3a (25%) or type 3b (6%) (Table 1).

Patient characteristics between the 44 participating and 16 non-participating individuals did not differ (gender: *p* = 0.639; age: *p* = 0.360).

### Questionnaires

#### Screening questionnaire:

Baseline and follow-up data on mandibular function are summarized in Table 2. Patients with limitations of mandibular function at baseline reported these limitations four years later. At follow-up, there was an increase of reported problems with mouth opening (30 vs 39%, respectively). Frequencies of mastication difficulties increased from 25% to 41% .

**Table 2:** Table2

Total (*n* = 44)			SMA type 2 (*n* = 22)			SMA type 3a (*n* = 12)			SMA type 3b (*n* = 10)		
	T1	T2	*p*-value	T1	T2	*p*-value	T1	T2	*p*-value	T1	T2	*p*-value
Mean age, yrs	33.4 (17.0)	37.3 (17.0)		27.4 (15.7)	31.2 (15.7)		36.1 (18.4)	40.2 (18.4)		43.3 (13.5)	47.2 (13.6)
Problems mouth opening^a^	13 (30)	17 (39)	0.2	12 (55)	14 (64)	0.6	1 (8)	2 (17)	1.0	0	1 (10)	–
Mastication difficulties^b^	11 (25)	17 (39)	0.1	7 (32)	9 (41)	0.6	3 (25)	4 (33)	1.0	1 (10)	4 (40)	0.4
Difficulties biting of food^c^	19 (43)	22 (50)	0.4	15 (68)	16 (73)	1.0	4 (33)	4 (33)	1.0	0	2 (20)	–
Food adaptation^d^	20 (45)	28 (66)	0.03	13 (59)	14 (64)	1.0	3 (25)	10 (83)	0.02	4 (40)	4 (40)	1.0
Physical therapy
None	9 (20)	10 (25)	–	5 (23)	5 (23)	–		2 (17)	–	4 (40)	3 (30)	–
Body	34 (77)	29 (64)		16 (73)	14 (64)		12 (100)	10 (83)		6 (60)	5 (50)
Body and orofacial		4 (9)			2 (9)						2 (20)
MFIQ raw score^e^	8.6 (10.8)	11.4 (12.2)	0.002	12.1 (12.8)	16.1 (13.8)	<0.01	7.2 (8.0)	9.3 (9.0)	0.476	2.1 (2.3)	3.3 (4.4)	0.385
AMMO ^f^(mm)	40.4 (15.5)	38.3 (17.1)	<0.001	29.2 (11.8)	25.7 (12.3)	<0.01	46.1 (6.9)	44.6 (8.2)	0.236	58.4 (7.1)	58.6 (7.4)	0.591
Lateral ROM^g^ L/R (mm)	8.3 (4.5)	7.6 (4.2)	0.041	6.0 (4.3)	4.6 (3.2)	0.003	9.0 (2.1)	8.8 (1.4)	0.633	12.4 (4.1)	12.5 (3.1)	0.929
Protrusion^h^ (mm)	6.8 (4.0)	5.9 (3.7)	0.038	5.0 (3.5)	3.9 ((3.5)	0.044	8.3 (3.7)	7.3 (2.5)	0.349	9.0 (3.5)	8.8 (2.5)	0.780
AMMO≤ 35 mm	17 (39)	21 (48)	0.125	15 (68)	18 (82)	0.250	2 (17%)	3 (25)	1.000	0	0
Limited sliding TMJ	10 (25)	19 (43)	0.004	10 (45)	17 (77)	0.016	0	2 (17)	–	0	0	–
Occlusal contacts	7.0 (1.7)	7.1 (1.7)	0.819	6.7 (2.2)	6.8 (2.0)	0.796	7.6 (0.8)	7.6 (1.2)	1.000	7.0 (1.3)	7.0 (1.3)	–
Anterior MVBF^i^ (N)	161.7 (76.7)	165.5 (82.6)	0.568	127.6 (67.7)	130.6 (62.5)	0.741	186.9 (73.2)	193.2 (100.6)	0.711	206.3 (70.5)	208.8 (71.0)	0.804
Mixing ability	18.2 (2.7)	18.5 (2.6)	0.377	19.2 (3.2)	19.6 (2.9)	0.607	17.2 (1.7)	17.0 (2.1)	0.706	17.2 (1.5)	18.2 (1.4)	0.136

^ *^Statistically significant differences; SMA = spinal muscular atrophy; ^a^Problems with mouth opening: rated on a 5-point scale. 0 = never; 1 = sometimes; 2 = regularly; 3 = often; 4 = very often. Patients with a score > 1 are considered to have difficulties with opening the mouth ^b^Mastication difficulties: rated on a 5-point scale. 0 = never; 1 = sometimes; 2 = regularly; 3 = often; 4 = very often. Patients with a score > 1 are considered to have mastication difficulties during mastication of hard, sticky or soft food; ^c^Difficulties biting of food: rated on a 5-point scale. 0 = never; 1 = sometimes; 2 = regularly; 3 = often; 4 = very often. Patients with a score > 1 have difficulties with biting of food; ^d^Food adaptation: patients unable to eat hard biscuits, meat, raw carrots, French loaf or nuts; ^e^MFIQ = Mandibular Functional Impairment Questionnaire. ^f^AMMO: active maximum mouth opening; ^g^Lateral ROM L/R: active mandibular lateral excursion; ^h^Protrusion: active forward movement of the mandible; ^i^MVBF: anterior maximum voluntary bite force. SMA type comparison and descriptive statistics at baseline in 2014 (T1) and follow-up in 2019 (T2). Mean and standard deviation of the measurements referred to as $\bar{x}$ (SD), number of patient as *n* (%).

Prevalence of self-reported dysphagia increased from 18 (41%) at baseline to 27 (62%) after four years (Table 1).

#### Mandibular Function Impairment Questionnaire

The MFIQ changed significantly over time at group level (*p* = 0.002). Subgroup analyses showed that the MFIQ increase, indicating increased mandibular problems at follow-up, was attributable to patients with SMA type 2 who had a mean score of 12.1 (SD 12.8) at baseline and 16.1 (SD 13.8) at follow-up (*p* < 0.001). For patients with SMA types 3a and 3b the MFIQ changes over time were not significant. Patients with SMA type 3a scored 7.2 (SD 8.0) vs 9.3 (SD 9.0) respectively (*p* = 0.476) and patients with type 3b scored 2.1 (SD 2.3) vs 3.3 (SD 4.4), respectively (*p* = 0.385).

### Clinical examination

#### Range of motion

**Table 3:** Table3

Univariate analysis		Multivariate analysis^a^			
	Effect size (β)	95% CI	*P*-value	Effect size (β)	95% CI	*p*-value
Age T1^b^	0.8 mm	0.2; 1.3	0.007	0.6 mm	0.0; 1.3	0.045
SMA type 2 vs type 3	–3.7 mm	–5.9; –1.5	0.002	–2.0 mm	–3.8; –0.1	0.043
Disease duration T1^b^	0.6 mm	0.1; 1.2	0.031	#excluded
AMMO T1	0.1 mm	0.0; 0.2	0.004	#excluded
Biteforce T1 (N)^c^	0.2 mm	0.0; 0.3	0.013	#excluded
Mixing abilityT1	–0.2 mm	–0.6; 0.2	0.268	–
MFIQ T1	–0.0 mm	–0.1; 0.0	0.224	–

Outcomes of multiple linear regression analysis. #excluded in multivariate analysis with forward method. –not included in multivariate analysis. ^a^with forward method. ^b^outcomes presented for 10 years increase in age and duration. ^c^outcomes presented for 10 N increase in biteforce. CI confidence interval. AMMO active maximal mouth opening. T1 = at baseline; SMA = spinal muscular atrophy; *N* = Newton; MFIQ = Mandibular Functional Impairment Questionnaire. Predictors for reduction of AMMO during follow-up in SMA patients.

We found a significant reduction of AMMO (*p* < 0.001), active lateral ROM (*p* = 0.003) and mandibular protrusion (*p* = 0.044) among patients with SMA type 2 at follow-up (Table 2).

In the total cohort, the median AMMO decrease at follow-up was 2.1 mm (95% CI: 1.1; 3.1, *p* < 0.001) after a median follow-up 3.9 years (IQR 3.8–4.0). In patients with SMA type 2, median AMMO reduction at follow-up was 3.5 mm (95% CI: 2.3; 4.7, *p* < 0.001) (Fig. 1), while AMMO reduction was not significant in SMA types 3a (1.5 mm (95% CI: –1.1; 4.1) *p* = 0.236) and 3b (–0.2 mm (95% CI: –1.0; 0.6) *p* = 0.591). Mean annual changes of AMMO in patients with SMA type 2, type 3a and type 3b were –0.9 mm (95% CI: –1.2; –0.6, *p* < 0.001), –0.4 mm (95% CI: –1.1; 0.3, *p* = 0.230) and +0.1 mm (95% CI: –0.2; 0.3, *p* = 0.585), respectively.

### Predictive factors for decreasing AMMO

Outcomes of univariate and multivariate analyses are shown in Table 3. Older age at first measurement and longer disease duration were associated with less pronounced decreases of AMMO during follow-up in the univariate analysis. A larger AMMO or higher MVBF at baseline were associated with less decrease in AMMO over time. Multivariate regression analyses showed that SMA type 2 was an independent predictor of more pronounced decreases in AMMO during follow-up, whereas older age was an independent predictor of a more limited decrease (e.g. decline of AMMO was present but less pronounced compared to the decline seen at younger age).

#### TMJ mobility

In patients with SMA type 2 sliding of the TMJ changed significantly from normal to limited sliding (*p* = 0.016), whereas in SMA type 3a and 3b there was no significant difference at follow-up. Among the 7 patients with SMA type 2 whose mandibular mobility changed from normal into limited sliding, AMMO decreased from 32.6 mm (95% CI: 25.1;41.0) to 28.6 mm (95% CI: 21.8;35.4). One patient with SMA type 3a developed limited TMJ sliding with an AMMO reduction from 45 mm to 32 mm. Sliding of the TMJ was normal in all patients with SMA type 3b (Table 2).

#### Pain

In two patients with SMA type 3b pain could be provoked by palpation of ≥1 of the chewing muscles (VAS 10/100 mm) at baseline and follow-up. In patients with SMA type 2 and 3a no pain could be provoked at baseline or follow-up.

#### Unchanged variables

In 4 years’ time occlusal contacts, MVBF and masticatory performance did not change significantly in the total cohort or in one of the subgroups (Table 2).

## Discussion

In this study we present longitudinal data on bulbar function in patients with SMA types 2, 3a and 3b. The data show differences in vulnerability of bulbar muscles, with significant changes in AMMO over time (which reflects function of lateral pterygoid muscle), but not in bite force or food mixing ability in SMA type 2. Decreasing AMMO over time is seen most often in patients with SMA type 2 with an estimated reduction of almost 1 mm per year. Multivariate analyses show that this particularly pertains to younger patients of this cohort, which supports the assumption that intervention strategies should start early.

In SMA, limited mandibular mobility is thought to be primarily caused by fatty infiltration of the lateral pterygoid muscles that mediate sliding of the condyles inside the TMJs, allowing opening of the mouth beyond the first 20 to 30 mm [4]. Our first study of AMMO in SMA was confined to patients with SMA type 2 [7]. In a larger cohort that included a broader spectrum of SMA severity, reduced AMMO was primarily, although not exclusively, associated with SMA types 1c and 2 and virtually absent in SMA type 3 [4]. In our current work we found that some patients with SMA type 3a may also experience AMMO limitations. Multivariate analyses suggest that the previously reported association with disease duration is not necessarily linear, and the rates of decline may be more pronounced at younger ages. Since the analysis was based on relatively few measurements in patients over 40 years, this needs to be corroborated in future studies.

At the more severe end of the SMA spectrum, mouth opening limitations may cause problems already at young ages, as exemplified by the finding that intubation was complicated or impossible in children with SMA types 1c and 2 who had to undergo scoliosis surgery [9]. In our work, the estimated reduction of AMMO was approximately 1 mm per year in patients with SMA type 2, although this may be an underestimation since more patients with type 2 than type 3 declined to participate in the follow-up study.

Reductions of AMMO are contrasted by the relatively stable values of biting force. The m. masseter and m. temporalis, which are mainly responsible for the bite force, therefore seem less affected than the m. pterygoideus lateralis that is responsible for mouth opening [4]. This suggests that some bulbar muscles are selectively vulnerable in SMA, similar to what is seen at other levels of the spinal cord. In the arms and legs, proximal muscles are usually more affected than the distal muscles, and the deltoid, triceps, iliopsoas and quadriceps muscles are weaker than biceps, hamstrings or gluteal muscles [26, 27]. Of the respiratory muscles, the diaphragm is relatively spared.

Results from the MFIQ showed a statistically significant increase in the percentage of patients with SMA type 2 experiencing problems at follow-up. The number of patients who had to adapt food increased, in particular among patients with SMA type 3a. These findings are not explained by limitations of AMMO because only two patients had a limited opening of respectively 34 mm and 35 mm. We hypothesize that fatigability of masticatory muscles explains the relatively high percentage of patients with SMA type 3a adapting food [10, 28, 29]. It is not surprising that the mixing ability did not change significantly in the entire cohort or for one of the SMA types, because of the unchanged occlusal contacts and the MVBF. Outcome of the mixing test is known to be primarily determined by the number of occlusal contacts and the MVBF [30]. Future studies should include tests that can capture fatigability of the masticatory muscles, such as the 6 minute chewing test [31].

Two patients with SMA type 3b reported pain during tests of mandibular function and palpation of ≥1 of the chewing muscles, both at baseline and at follow-up. A recent study that assessed complaints of pain in SMA, using the 36-item Short Form Health Survey (SF-36), showed that pain was reported more often by patients with later-onset forms of SMA (types 3b and 4) compared to those with earlier-onset SMA (type 1, 2, 3a) [32]. The etiology of pain in SMA is multifactorial and, among other factors, may include spinal deformities, muscle cramps, or neurogenic pain [33]. To the best of our knowledge, there are no reports of masticatory muscle pain as a disease characteristic of SMA. Masticatory muscle pain in SMA may therefore have been observed coincidentally. Our findings are in the line with findings from a national survey in The Netherlands, in which 5% of the healthy adult population reported to perceive some dysfunction of the masticatory system, and 44.4% showed signs and symptoms of a temporomandibular disorder, a musculoskeletal condition that involves the masticatory musculature, the TMJ, associated structures, or a combination [34].

At present it is not known if therapies for SMA may prevent a reduction of AMMO. Although physical therapy is part of the proactive supportive care recommended for patients with SMA, no specific recommendations regarding orofacial therapy are available [35].

**Fig. 2:**
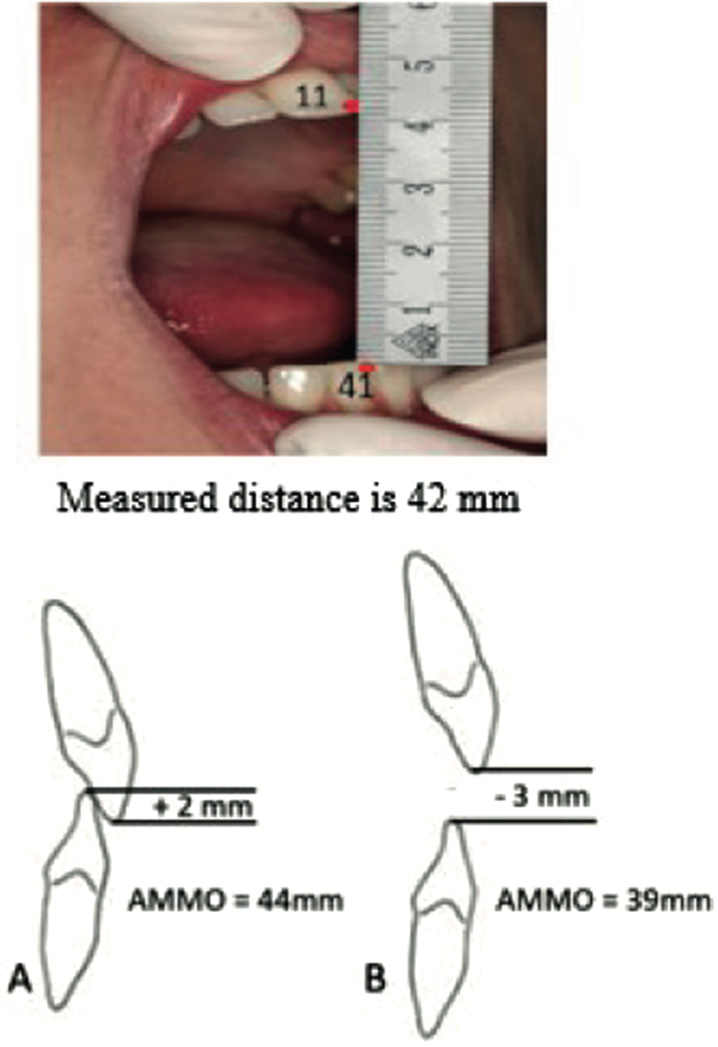
Protocol to measure the maximum mouth opening with a ruler. Verbal instruction: After opening the mouth widely several times the following instruction was given: “open as wide as possible, wider, wider’. Measurement: Ruler from the incisal edge of the right maxillary central incisor tot the incisal edge of the right mandibular central incisor at the midline and measure the distance. Correction for tooth position: Add up the overbite (A) or subtract the open bit (B).

Measuring AMMO with a ruler is easy, reproducible and reliable (Fig. 2) [17, 36]. Correction for vertical tooth position by extracting a positive overbite value or subtracting a negative overbite value from the measured AMMO is important, because abnormal craniofacial growth patterns and dental malocclusions in patients with SMA may occur [11]. Disposable paper rulers are probably an even simpler methodology.

The natural history data on AMMO provided in our work may be of value to assess the efficacy of new (genetic) treatments at the level of the brainstem. Future research should also address effectiveness of specific orofacial training programs for patients with SMA. At this point, we propose that measurements and documentation of AMMO are performed routinely as part of the care provided to patients with SMA. Since AMMO measurement is easy and reliable to perform, it can be used to foresee intubation difficulties that can occur in SMA [9]. As a part of the proactive supportive care, frequent orofacial exercises should probably be initiated at an early age, before limitations of AMMO are present. This may include passive stretching of mouth opening and passive movement of the mandible in all horizontal directions. Starting exercises early, while individuals still have mobile TMJs and fatty infiltration of muscle tissue is not yet advanced, will probably be most effective.

## DATA AVAILABILITY STATEMENT

The data supporting the findings of this study are available on reasonable request from the corresponding author. The data are not publicly available due to privacy or ethical restrictions.

## STATISTICAL ANALYSIS

A. Ferdows.

Department of Hospital Pharmacy, Erasmus Medical Center, Rotterdam, The Netherlands.

## AUTHOR CONTRIBUTIONS

van Bruggen HW: obtaining funding, drafting the manuscript for content, including writing for content, study concept or design, analysis or interpretation of data, acquisition of data, statistical analysis.

Wijngaarde CA: revising the manuscript for content, including writing for content, acquisition of data, analysis or interpretation of data.

Asselman F: revising the manuscript for content, including writing for content, acquisition of data.

Stam M: revising the manuscript for content, including writing for content, acquisition of data, analysis or interpretation of data.

Creugers NHJ: revising the manuscript for content, including writing for content.

van der Pol WL: obtaining funding, study concept or design, revising the manuscript for content, including writing for content, analysis or interpretation of data.

Wadman RI: revising the manuscript for content, including writing for content, acquisition of data, analysis or interpretation of data.

Kalaykova SI: obtaining funding, study concept or design, revising the manuscript for content, including writing for content, analysis or interpretation of data.

All authors gave their final approval and agree to be accountable for all aspects of the work.
